# Reversal of the Progression of Fatal Coronavirus Infection in Cats by a Broad-Spectrum Coronavirus Protease Inhibitor

**DOI:** 10.1371/journal.ppat.1005531

**Published:** 2016-03-30

**Authors:** Yunjeong Kim, Hongwei Liu, Anushka C. Galasiti Kankanamalage, Sahani Weerasekara, Duy H. Hua, William C. Groutas, Kyeong-Ok Chang, Niels C. Pedersen

**Affiliations:** 1 Department of Diagnostic Medicine and Pathobiology, College of Veterinary Medicine, Kansas State University, Manhattan, Kansas, United States of America; 2 Department of Medicine and Epidemiology, School of Veterinary Medicine, University of California at Davis, Davis, California, United States of America; 3 Department of Chemistry, Wichita State University, Wichita, Kansas, United States of America; 4 Department of Chemistry, Kansas State University, Manhattan, Kansas, United States of America; University of Iowa, UNITED STATES

## Abstract

Coronaviruses infect animals and humans causing a wide range of diseases. The diversity of coronaviruses in many mammalian species is contributed by relatively high mutation and recombination rates during replication. This dynamic nature of coronaviruses may facilitate cross-species transmission and shifts in tissue or cell tropism in a host, resulting in substantial change in virulence. Feline enteric coronavirus (FECV) causes inapparent or mild enteritis in cats, but a highly fatal disease, called feline infectious peritonitis (FIP), can arise through mutation of FECV to FIP virus (FIPV). The pathogenesis of FIP is intimately associated with immune responses and involves depletion of T cells, features shared by some other coronaviruses like Severe Acute Respiratory Syndrome Coronavirus. The increasing risks of highly virulent coronavirus infections in humans or animals call for effective antiviral drugs, but no such measures are yet available. Previously, we have reported the inhibitors that target 3C-like protease (3CLpro) with broad-spectrum activity against important human and animal coronaviruses. Here, we evaluated the therapeutic efficacy of our 3CLpro inhibitor in laboratory cats with FIP. Experimental FIP is 100% fatal once certain clinical and laboratory signs become apparent. We found that antiviral treatment led to full recovery of cats when treatment was started at a stage of disease that would be otherwise fatal if left untreated. Antiviral treatment was associated with a rapid improvement in fever, ascites, lymphopenia and gross signs of illness and cats returned to normal health within 20 days or less of treatment. Significant reduction in viral titers was also observed in cats. These results indicate that continuous virus replication is required for progression of immune-mediated inflammatory disease of FIP. These findings may provide important insights into devising therapeutic strategies and selection of antiviral compounds for further development for important coronaviruses in animals and humans.

## Author Summary

Coronaviruses are important pathogens in humans and animals. Although some coronaviruses can cause severe illness in humans and animals with considerable fatality, there is no antiviral drugs available for coronavirus infections. Feline infectious peritonitis (FIP), caused by virulent feline coronavirus, is the leading infectious cause of death in young cats, and also threatens endangered captive wild cats. We have previously reported series of small molecule protease inhibitors with broad-spectrum activity against important human and animal coronaviruses. In this report, we provide, for the first time, experimental evidence of efficacy and safety of one of the protease inhibitors in laboratory cats with experimentally induced FIP. These findings suggest that direct inhibition of virus replication by a protease inhibitor can be devised as a viable treatment option for coronavirus infection and our protease inhibitor has a potential to be developed into an effective therapeutic agent for FIP.

## Introduction

Coronaviruses comprise a large family of RNA viruses that infect a wide variety of mammalian and avian hosts causing a broad spectrum of diseases. Coronaviruses have a single-stranded, positive-sense RNA genome and are classified into four genera of *alpha-*, *beta-*, *gamma-*, and *deltacoronaviruses* [1]. Coronaviruses are prone to mutation and recombination during replication and this propensity has contributed to the existing diversity of coronaviruses [2, 3]. Sudden emergence of new coronaviruses transmitted from animal hosts, Severe Acute Respiratory Syndrome Coronavirus (SARS-CoV) and, more recently, Middle East Respiratory Syndrome Coronavirus (MERS-CoV), has raised awareness about the potential risks of highly virulent coronavirus infections in humans with increasing close contact between humans and animals harboring coronaviruses. However, effective therapeutic measures for coronavirus infections have been elusive so far despite the extensive efforts in the development of anti-coronavirus agents [4–8]. Shifts in tissue or cell tropism and resulting changes in virulence have also been reported for coronaviruses; porcine respiratory coronavirus causes mild respiratory infection in pigs and presumably arose from transmissible gastroenteritis virus (TGEV), the etiologic agent of gastroenteritis in young pigs with a high fatality, by spontaneous mutations and/or deletions in its genome [9]. Seemingly innocuous coronavirus infection can also be turned deadly by changing its tropism, exemplified by mutation of feline enteric coronavirus (FECV) to feline infectious peritonitis virus (FIPV) [10, 11]. Feline infectious peritonitis (FIP) has intrigued researchers for half a century since its first description in the 1960s [10]. Infection with FECV which causes inapparent or mild enteritis is widespread among cats, especially in high-density environments, and has little clinical consequence. However, a small portion of cats develop FIP during the course of FECV infection and succumb to the disease. Published studies support that FIP arises in individual cats through mutation of the virus to gain tropism for macrophages [12–16] and that the immune system of the infected cats plays an important role in the pathogenesis of FIP [11]. FIP occurs in two major forms, effusive (wet) form or non-effusive (dry) form. The wet form is more common (60–70% of FIP cases) and characterized by accumulation of fluids in the abdominal and/or, to a lesser degree, chest cavities [11]. Granulomatous vasculitis is frequently found in the omentum, mesenteric lymph nodes, and serosal surface of the large intestine, resulting in the characteristic exudates rich in protein and inflammatory cells in the body cavities in wet FIP [11]. The majority of exudate cells are virus-infected macrophages and high virus load is detected in these cells [17]. Multiple granulomatous lesions composed of macrophages laden with viruses and other inflammatory cells typically form in various tissues and organs, such as the omentum, mesenteric lymph nodes, spleen and liver, in both forms of FIP [17]. Clinical symptoms of FIP reflect the organs involved and include fever, jaundice, bodily effusions and weight loss and may also affect the central nervous system and the eyes [11].

Virus-induced immunopathogenesis and lymphopenia in cats with FIP are features also frequently associated with other coronavirus infections, such as SARS and MERS in humans. The causes for lymphopenia observed in these coronavirus infections are not fully elucidated but the published reports support that lymphopenia is related to the indirect effects of virus infection [18–20]. Lymphopenia associated with massive apoptosis of uninfected T-cells is a prominent feature of both experimental and natural FIP [11, 17, 21, 22] and implicated with cytokines secreted by the virus-infected macrophages and other immune cells [17, 19]. Lymphopenia precedes the onset of clinical signs and is associated with disease progression and death in experimental FIP, which indicates that impaired cellular immune responses associated with lymphocyte depletion is important in FIP pathogenesis [17, 22]. Once cats develop classic clinical signs, fatality to FIP is virtually 100% [17, 23–25] and the median survival time from the time of diagnosis to death or euthanasia is about 8–9 days [23, 24]. FIP is a leading cause of death among young cats under 2 years of age and estimated to kill 1 in 100 to 300 cats worldwide [10, 26]. FIP also affects endangered exotic cats in zoos, such as jaguars and cheetahs [27]. However, vaccines have proven ineffective and treatment is only palliative [25].

Studies of anti-coronavirus drugs have mainly focused on the discovery of anti-SARS-CoV agents. Effective treatment intervention for coronavirus infections with an immunopathological component, such as SARS, MERS and FIP, is speculated to involve the judicious use of immunomodulatory agents to enhance protective host immunity and decrease pathological immune responses and antiviral drugs to directly inhibit viral replication. We have previously reported several series of small synthetic peptidyl compounds that target a virally-encoded protease, 3C-like protease (3CLpro) [28–30]. Coronavirus 3CLpro and papain-like protease (PLP) process viral polyproteins into functional individual proteins and their structures are highly conserved among coronaviruses. Since viral proteases are indispensable for virus replication, many synthetic small molecules or natural compounds targeting 3CLpro or PLP of coronaviruses have been investigated using the *in vitro* systems [4–8]. However, only few studies tested the *in vivo* efficacy of protease inhibitors in experimental animals [30, 31]. Deng et at [31] reported that a PLP inhibitor failed to reduce virus titers in the lung or increase the survival of mice infected with a mouse-adapted SARS-CoV, presumably due to low bioavailability or stability of the inhibitor. Recently, we demonstrated that our 3CLpro inhibitors significantly decreased the virus titers and pathology in the liver of mice infected with murine hepatitis virus (MHV), a murine coronavirus [30]. In those studies, treatment was started shortly before or after virus infection in asymptomatic mice.

Here we extended our previous work on coronavirus 3CLpro inhibitors and investigated the pharmacokinetics (PK), safety and efficacy of a 3CLpro inhibitor in cats. GC376 is a 3CLpro inhibitor which is previously reported to be active against the 3CLpro of multiple coronaviruses, including SARS-CoV [28], but with highest potency against FIPV in cell culture. In this study, we determined that GC376 exhibited favorable bioavailability and safety in cats. In the *in vivo* efficacy study using GC376 in cats experimentally infected with FIPV, antiviral treatment was started after the cats reached a clinical stage that would ultimately lead to death, if untreated. Antiviral treatment caused a rapid reversal of clinical signs and lymphopenia and reduction in viral titers in the macrophages from the ascites. Active infection was no longer apparent after 14–20 days of antiviral treatment and the treated cats have remained normal under observation for as long as eight months. These results provide important first evidence that a 3CLpro inhibitor is effective at reversing disease progression when administered to cats in an advanced and invariably fatal stage of experimentally induced FIP.

## Results

### Pharmacokinetics study of GC376 in cats

**Fig 1:**
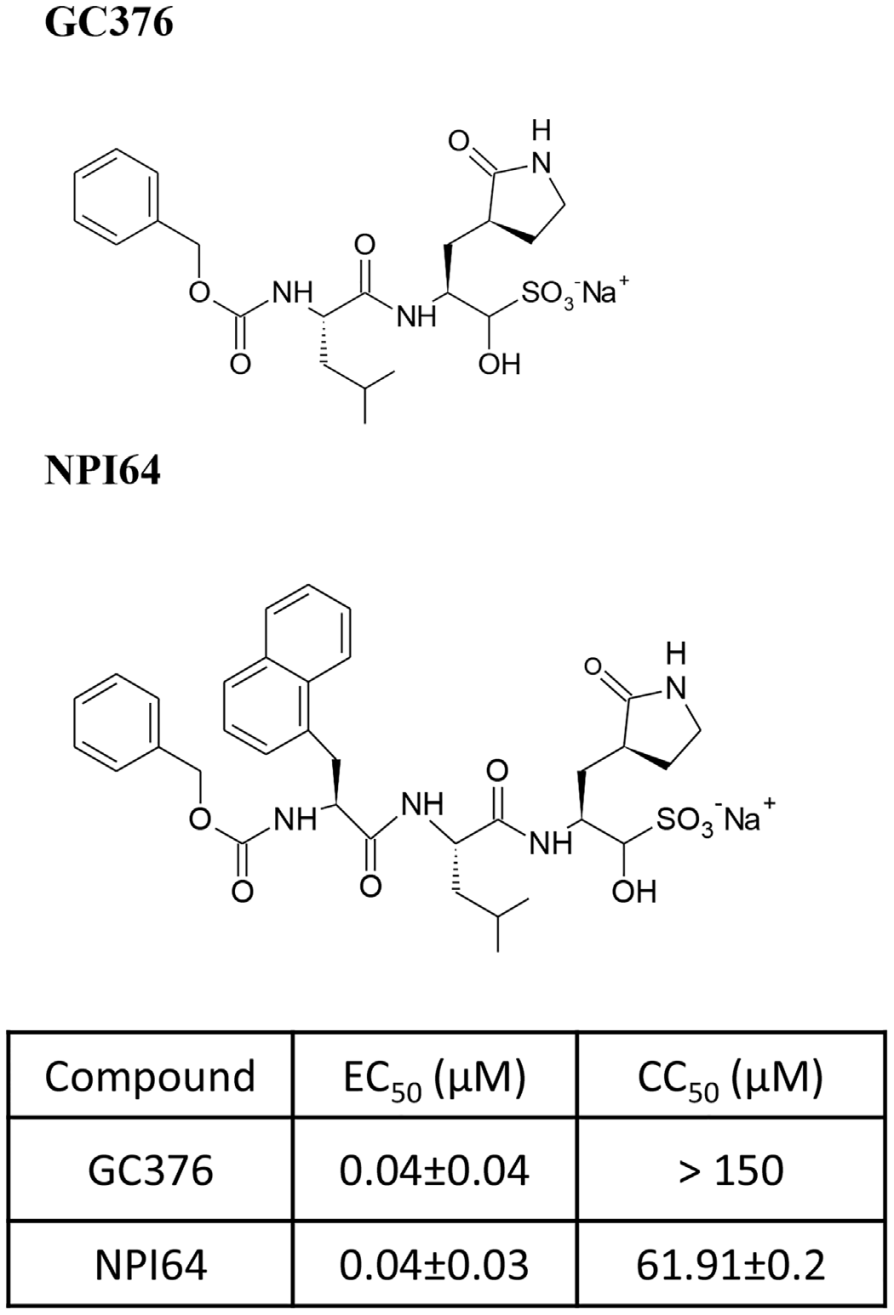
The chemical structures of 3CLpro inhibitors and their antiviral activity against feline coronavirus in cell culture. The chemical structures of GC376 and NPI64 are shown. The 50% effective concentration (EC_50_) values of GC376 or NPI64 against FIPV 3CLpro [28, 30] and the 50% cytotoxic concentration (CC_50_) values of GC376 or NPI64 determined in various cell lines were previously reported [28, 30] and summarized in a table.

**Fig 2:**
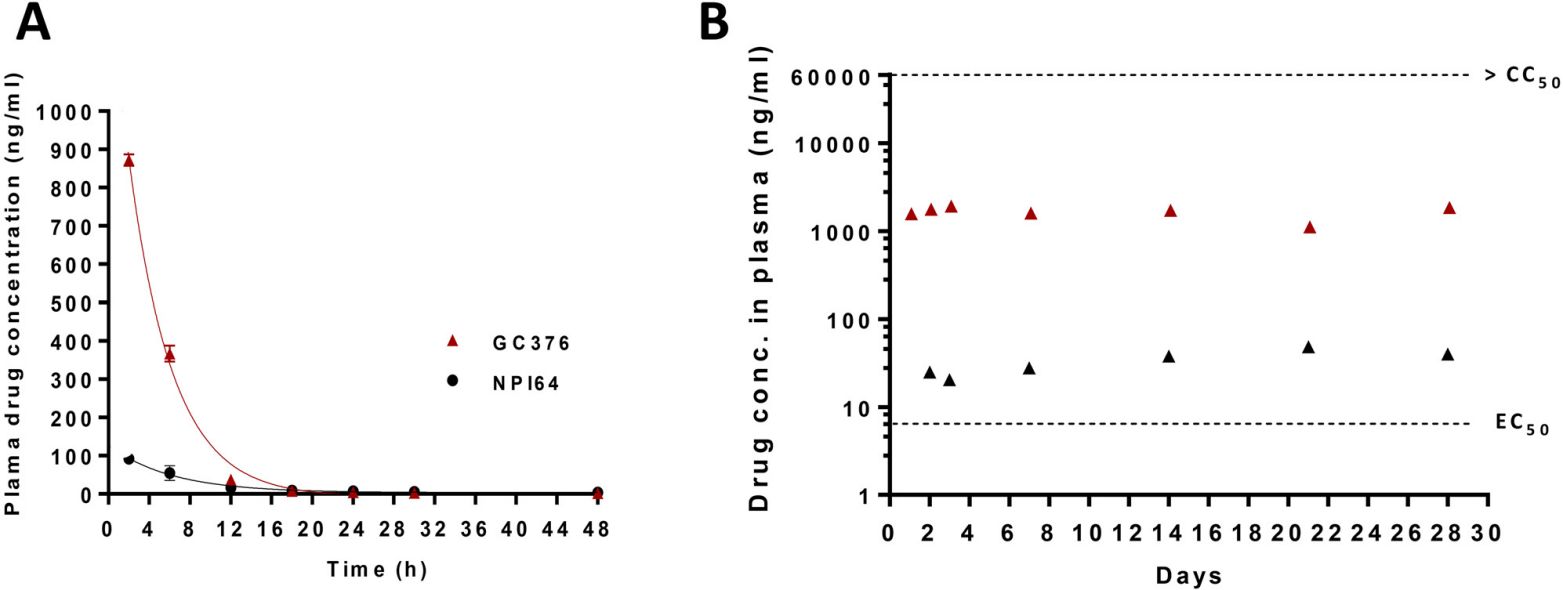
Changes in plasma drug concentrations after administration of 3CLpro inhibitors via a subcutaneous route. (A) In the single-dose pharmacokinetics study, two healthy specific pathogen free (SPF) cats were subcutaneously injected with GC376 at 10 mg/kg/dose or NPI64 at 5 mg/kg for the determination of serial plasma drug concentrations. GC376 and NPI64 are readily converted into aldehyde forms in the blood [28]. The red triangles and black circles indicate the plasma concentrations of the aldehyde forms of GC376 and NPI64, respectively (means and standard error of the means are shown). (B) In the safety study, four healthy SPF cats were subcutaneously given GC376 at 10 mg/kg/dose daily at 9 AM and 5 PM for 4 weeks. During that time, plasma drug concentrations were measured at 2 and 16 hr post-injection for the first three days and weekly thereafter (red and black triangles, respectively, means are shown). The dotted red line indicates the EC_50_ value of GC376. The 50% cytotoxic concentration (CC_50_) value of GC376 (>150 μM) is greater than the dotted blue line.

GC376 (Fig 1) is a representative compound of the dipeptidyl transition state 3CLpro inhibitors [28–30, 32] whose synthesis was described previously [28]. NPI64 shares homologous structural elements with GC376, except that NPI64 has an additional residue of 1-naththylalanine compared to GC376 in a position that corresponds to the P3 position [30], using the nomenclature of Schechter and Berger [33] (Fig 1). The comparable antiviral activity of GC376 and NPI64 against the replication of feline coronavirus in a cell culture system was previously reported (Fig 1) [28, 30]. However, their PK properties have not been reported. In this study, we investigated the drug plasma concentration changes in healthy specific pathogen free (SPF) cats of 6–9 month age (n = 2 for each compound) following single subcutaneous (s.c.) dose of 10 mg/kg GC376 or 5 mg/kg NPI64. Serial blood samples were then collected and the plasma drug concentrations were measured. Previously, we reported that GC376 is converted into an aldehyde form by the removal of the bisulfite group, and the aldehyde form forms a reversible covalent bond with the nucleophilic cysteine residue of 3CLpro in the x-ray crystallography studies [28]. We also observed the conversion of NPI64 into its aldehyde form in the blood. Therefore the aldehyde forms of GC376 or NPI64 were measured in the plasma samples. Fig 2A shows the plasma drug concentrations over time following single-dose administration of GC376 (red triangles) or NPI64 (black circles). The PK study results indicate that GC376 is rapidly absorbed after s.c. administration and the peak plasma level was reached within 2 hr after injection. The mean plasma drug concentrations remained above the 50% effective concentration (EC_50_) value of the aldehyde form of GC376 (8 ng/ml) for 18 hrs post injection (Fig 2A, red triangles). The plasma drug concentrations following injection of 5 mg/kg NPI64 stayed above the EC_50_ value of the aldehyde form of NPI64 (12 ng/ml) for 12 hrs post injection (Fig 2A, black circles). The maximum detected plasma drug concentration following NPI64 administration was substantially lower than that of GC376 by 9.5-fold. This result indicate that GC376 was more easily absorbed than NPI64 via the tested route, even when the lower dose of NPI64 (5 mg/kg), compared to GC376 (10 mg/kg), was taken into account.

### Four-week safety study of multiple doses of GC376 in cats

After the dosage regimen of GC376 was determined in the PK study, safety of GC376 was evaluated in four healthy SPF cats of 6–9 months of age. The cats were administered with 10 mg/kg GC376 by s.c. injection twice daily at 9 AM and 5 PM for 4 weeks. For the duration of the study, they were observed daily for adverse effects. Blood samples were taken weekly and the complete blood counts and blood chemistry panels were conducted. During the study period, there were no clinically significant changes in vital signs and clinical lab parameters (S1A–S1D Fig), indicating that the dosage and the route of administration of GC376 was well-tolerated in cats for the duration of the safety study.

During the safety study, additional blood samples were taken at 2 and 16 hr post drug administration for the first three days and then weekly for 4 weeks. The plasma drug concentrations at 16 hr post administration were determined from the blood collected immediately before next drug administration and thus represent the minimum drug levels in the plasma. The results show that the lowest plasma drug concentrations remained above the EC_50_ value (Fig 2B black triangles) and that the highest determined drug concentrations were well below the CC_50_ value which is greater than 150 μM in cell culture [30] (Fig 2B red triangles). Based on the results from the safety and the PK studies, the dose and administration route of GC376 was determined to be suitable for the *in vivo* efficacy study.

### Experimental infection of cats with FIP and antiviral treatment

The experimental infection of cats with serotype I FIPV that induces wet FIP has been reported previously [12, 17, 34]. FIPV is classified into serotypes I and II based on virus neutralization tests. Serotype I FIPV is responsible for the majority (80–90%) of naturally-occurring FIP [10, 35–38]. In this experimental infection, an absolute lymphopenia, fever, weight loss, jaundice and inapparent to mild ascites appear within 2–3 weeks after infection. Increasing jaundice and ascites occur during the next 1–3 weeks. All the cats that develop lymphopenia and clinical signs following experimental infection do not spontaneously recover but succumb to the disease [12, 17, 34].

**Fig 3:**
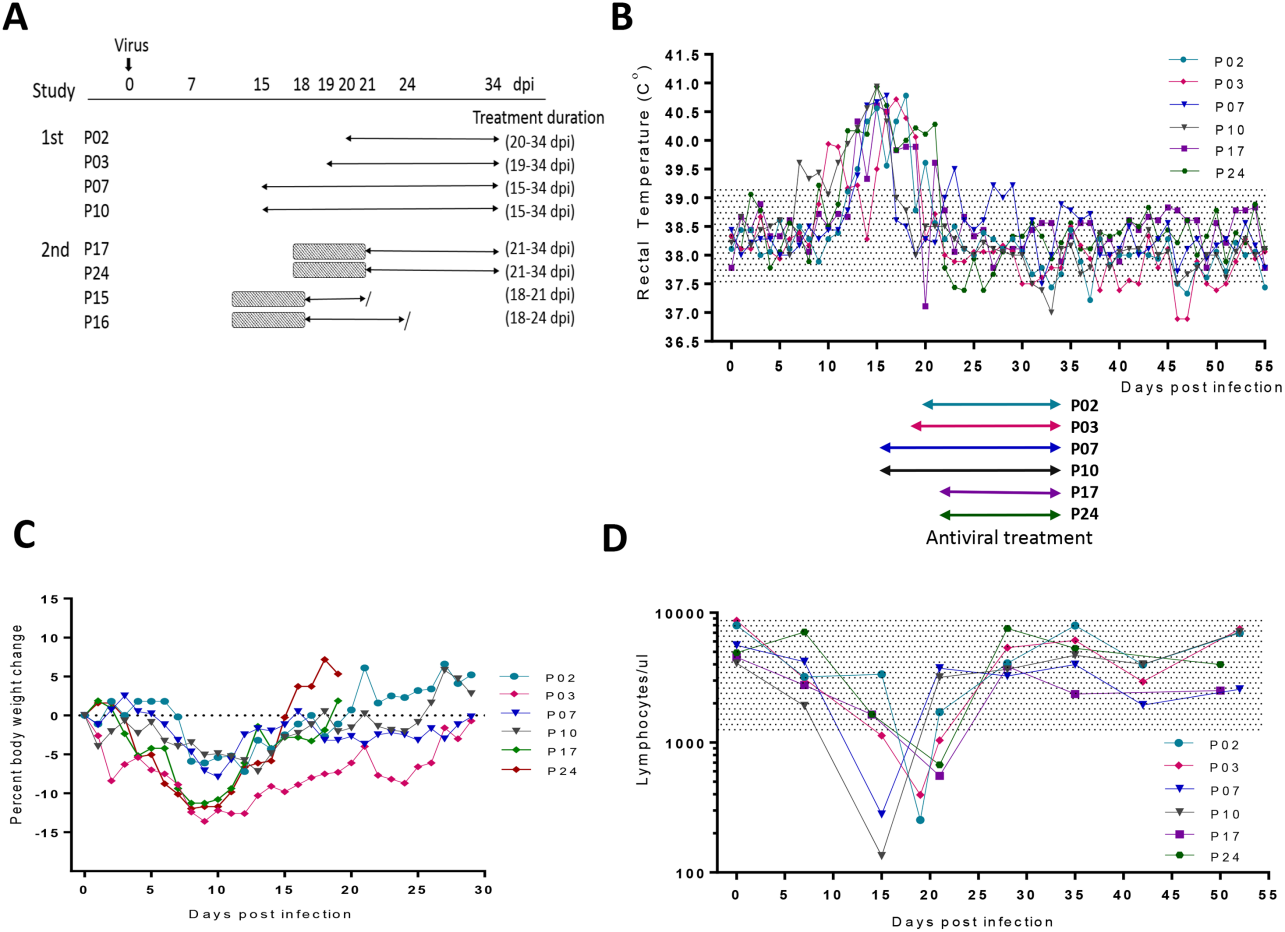
Antiviral treatment of symptomatic cats with FIP. (A) In two independent studies, cats were inoculated with FIPV at day 0 and GC376 treatment was started after they developed lymphopenia and clinical symptoms. In the 2^nd^ study, cats received supportive treatment for five days (shaded boxes), which was discontinued prior to antiviral treatment. The arrows and forward slashes indicate antiviral treatment duration and euthanasia, respectively. dpi, days post infection. (B-D) Responses of cats with FIP to antiviral treatment: body temperature (B), percent body weight changes (C) and lymphocyte counts (D) over time. The shaded areas indicate the normal range of values. Colored arrows located between panels B and D indicate the treatment duration for each cat.

To investigate the efficacy of GC376, we conducted two independent studies. In these studies, antiviral treatment was started after the infected cats developed the typical laboratory finding of absolute lymphopenia and clinical symptoms to determine whether treatment with GC376 is effective in reducing the severity of symptoms or fatality. In both studies, the infected cats were monitored daily for fever, body weight, and outward disease signs and weekly for lymphocyte counts. In the first efficacy study, four SPF cats of 8–10 months of age (P02, P03, P07 and P10) were intraperitoneally administrated with a cat-passaged serotype I FIPV (FIPV-m3c-2) [12, 17, 34]. Following infection, they developed lymphopenia and clinical symptoms including inapparent or mild ascites within 14–20 days post infection (dpi) (Table 1). In the second study, the ascites of four SPF cats of 8–10 months of age inoculated with the same virus (P15, P16, P17 and P24) were allowed to progress to more profound, classical abdominal effusions, which closely resemble those of cats with naturally-occurring FIP frequently presented at the clinics (Table 1). However, in order to alleviate suffering, the latter four cats were given meloxicam, a non-steroidal anti-inflammatory drug, and subcutaneous fluids prior to antiviral treatment. This supportive treatment was discontinued before antiviral drug treatment commenced. The eight cats from both studies developed jaundice, inapparent to profound ascites, absolute lymphopenia (134~676/μl, reference range 1,200 to 8,000/μl) and high fever (up to 41.1°C) (Fig 3B and 3D, Table 1) before antiviral treatment was started. They also lost body weight up to 13.6% of their pre-infection weight during this same period (Fig 3C). When they reached this stage, twice daily s.c. administration of GC376 at 5–10 mg/kg/dose was started. These cats were treated for 14–20 days, except for P15 and P16 that were euthanized after 4 and 7 days after starting antiviral treatment based on the severe nature of their clinical signs (Fig 3A). All six remaining cats showed rapid improvement in attitude and resolution of fever (Fig 3B). The profound absolute lymphopenia observed in all cats prior to antiviral treatment also returned to normal before the next blood testing one week later (Fig 3D) and weight losses were reversed and normal growth resumed (Fig 3C). Ascites and scrotal swelling indicative of peritonitis also gradually resolved after a week of antiviral treatment. All cats that received antiviral treatment for 14–20 days appeared normal by clinical observation and laboratory testing. The six recovered cats from both studies have remained healthy showing no signs of relapse during an observation period up to 8 months. These experiments demonstrate that the protease inhibitor was able to reverse disease progression when treatment was initiated at advanced clinical stages of FIP.

**Table 1:** Clinical and laboratory findings in cats challenged with FIPV prior to antiviral treatment.

	Clinical and laboratory findings prior to treatment						
Cat	Fever	Weight loss	Jaundice (mucous membrane or plasma)	Ascites	Lymphopenia (<676/μl)	Treatment duration (dpi)*	Outcome
P07	+	+	+	Not apparent	+	20 days (15–34)	Recovered
P10	+	+	+	Not apparent	+	20 days (15–34)	Recovered
P02	+	+	+	Mild	+	15 days (20–34)	Recovered
P03	+	+	+	Mild	+	16 days (19–34)	Recovered
P17	+	+	+	Profound	+	14 days (21–34)	Recovered
P24	+	+	+	Profound	+	14 days (21–34)	Recovered
P15	+	+	+	Profound	+	4 days (18–21)	Euthanized
P16	+	+	+	Profound	+	7 days (18–24)	Euthanized

* dpi, days post infection

### Antiviral treatment significantly reduced viral load in the cats with FIP

**Fig 4:**
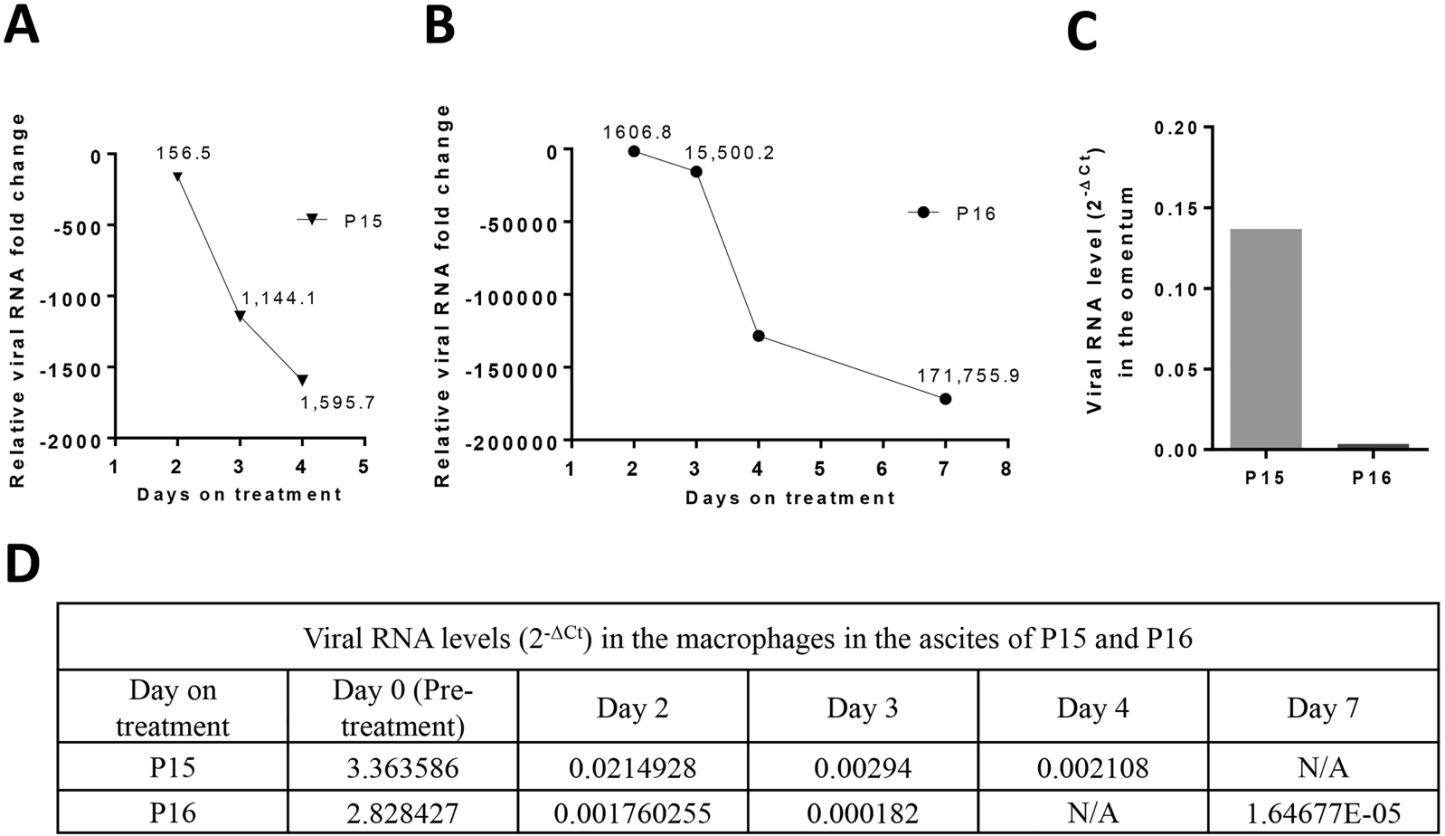
Changes in the viral RNA levels in P15 and P16 before and during antiviral treatment. (A and B) The viral RNA fold changes in the macrophages from the ascites of P15 (A) and P16 (B) over time are shown. The Ct values from viral RNA real-time qRT-PCR were normalized to β-actin and the 2^-ΔΔCt^ method was used to calculate the relative change in viral RNA level, compared to the pre-treatment value. (C) The viral RNA level (2^-ΔCt^) in the omentum of P15 and P16 which are collected after 4 and 7 days of antiviral treatment, respectively. The bar graph shows the 2^-ΔCt^ values calculated by normalizing the Ct values from viral RNA real-time qRT-PCR to β-actin. (D) The 2^-ΔCt^ values for each viral RNA in the macrophages from the ascites of P15 and P16 at pre-treatment and during treatment are listed in the table. N/A, not available.

Since FIPV is highly associated with tissues and is not reliably detected in blood at high levels in cats with FIP [17], assessment of the efficacy of antiviral drugs in reducing the viral load poses a difficulty in live animals. Although measuring virus titers of the exudate macrophages from the ascites allows to determine the effects of antiviral drug against the replication of FIPV, ascites rapidly decreased with antiviral treatment and we were not able to collect ascites in the recovered cats. However, we determined the viral load in two cats from the second study (P15 and P16) prior to and during antiviral treatment. These cats were euthanized after 4 and 7 days of antiviral treatment. On necropsy, both cats had severe pancreatitis, a possible complication of meloxicam treatment, but no lesions (P16) or mild lesions (P15) typical of FIP were found. Virus titers in the macrophages from the ascites were determined by real-time quantitative RT-PCR and the Ct values were analyzed by the comparative Ct method using the β-actin as a reference gene [39]. The results showed that viral RNA level in the macrophages from the ascites decreased commensurately with the duration of antiviral treatment in these cats. The fold reduction of viral RNA level determined using the delta delta Ct method was 1,595.7 in P15 that received 4 day-antiviral treatment (Fig 4A) and 171,755.9 in P16 that received 7 day-antiviral treatment (Fig 4B), compared to the pre-treatment viral RNA level in the macrophages of each cat. The viral RNA levels (2^-ΔCt^) in the macrophages from the ascites are summarized in Fig 4D. The viral RNA level in the omentum of P15 and P16 is also shown in Fig 4C. Based on these results, the reduction in virus titers in P15 and P16 seems to correlate with the necropsy findings of mild or no FIP lesions in those cats. These results on viral titers show that FIPV 3CLpro is a valid target for FIPV antiviral drugs and GC376 can effectively reduce the virus load in the macrophages from the ascites and the omentum of cats with FIP.

### Comparison of *in vitro* selection of GC376 and NPI52-resistant FIPV

Serial passages of FIPV-1146 in Crandell Rees feline kidney (CRFK) cells in the presence of GC376 or NPI52 (an aldehyde form of NPI64) were conducted to compare the emergence of viral resistance under drug pressure. At passage number 10, the EC_50_ value of NPI52 against FIPV increased by 15-fold, compared to wild-type virus at the same passage number. However, a decrease in antiviral activity of GC376 against FIPV was not observed at up to 20 passages. The sequence analysis of the 3CLpro gene of NPI52-resistant FIPV viruses collected from passage 10 revealed a single mutation of serine to cysteine at the position of 131, which is located between the β-strands cII and dII in the domain II (S2A Fig). Since these compounds share similar structure, we also investigated whether NPI52-resistant viruses retain susceptibility to GC376. GC376 effectively inhibited the replication of NPI52-resistant viruses in cell culture as wild-type viruses, indicating that the mutation does not confer cross-resistance to GC376.

### The 3CLpro inhibitor, GC376, is active against 3CLpro of MERS-CoV and SARS-CoV in a fluorescence resonance energy transfer (FRET) assay

**Fig 5:**
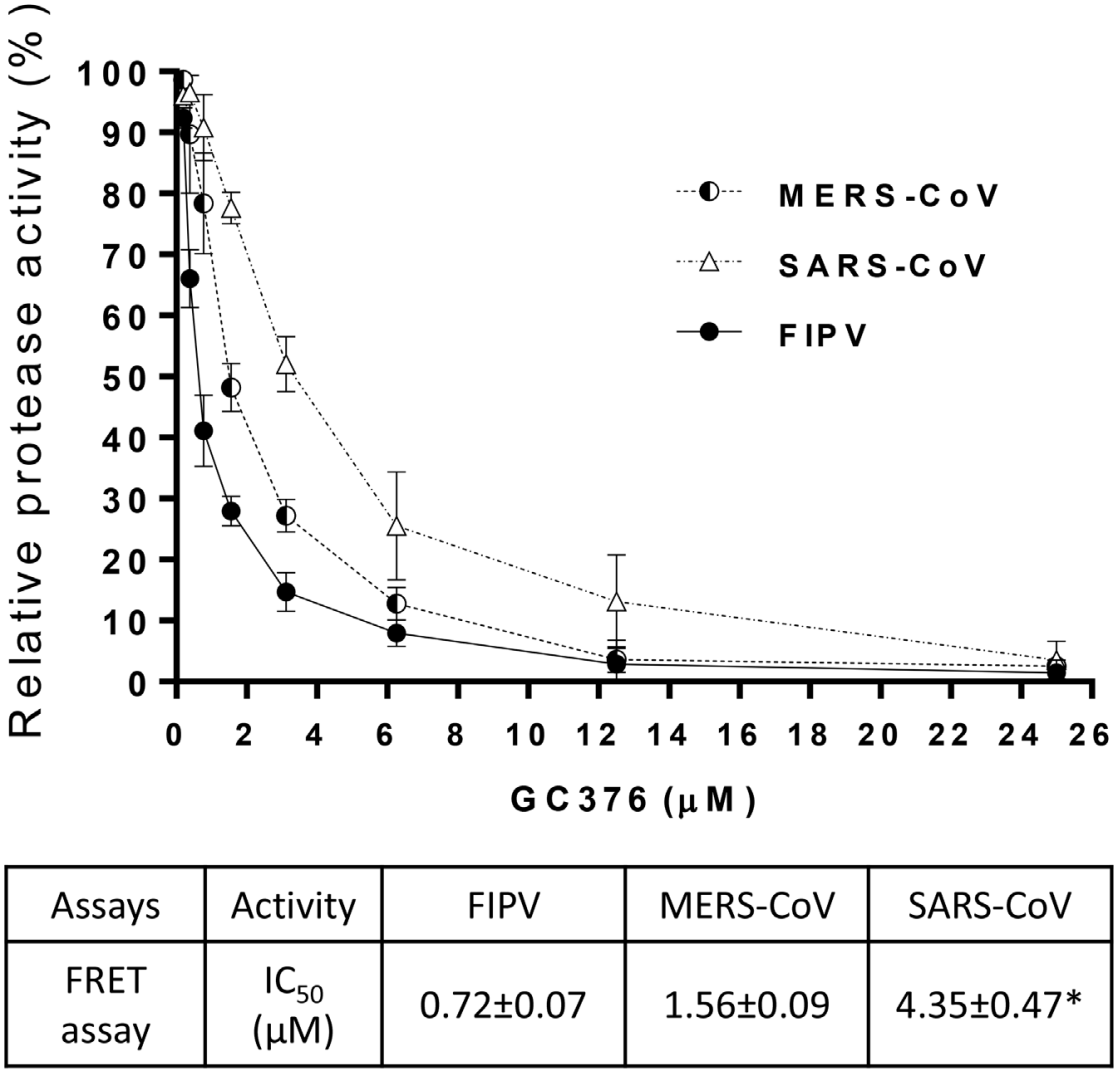
Activity of GC376 against 3CLpro of various coronaviruses in a fluorescence resonance energy transfer (FRET) assay. The upper graph shows the percent activity of 3CLpro of FIPV, SARS-CoV, and MERS-CoV in the presence of GC376, determined by a FRET assay. The lower table summarizes the 50% inhibitory concentration (IC_50_) values of GC376 against 3CLpro of FIPV, SARS-CoV, and MERS-CoV. Asterisks indicate the previously published value [28].

Coronavirus 3CLpro are highly conserved in their structure and the active site [28, 40, 41]. S2A Fig shows the superimposed 3CLpro structures of MERS-CoV (PDB ID: 4WME, teal) [40] and FIPV (red) modelled based on TGEV 3CLpro (PDB ID: 4F49) [28]. The 3CLpro of TGEV and FIPV are highly conserved with the amino acid sequence identity of >93%. However, 3CLpro of TGEV, MERS-CoV and SARS-CoV have low amino acid sequence identity of about 50%. Nonetheless, they share well conserved overall structure (S2A Fig). The activity of GC376 was previously reported against the 3CLpro of SARS-CoV using a FRET assay [28]. However, its activity against the 3CLpro of MERS-CoV and FIPV is unknown. Therefore, we cloned and expressed the full-length 3CLpro of FIPV and MERS-CoV following the procedures described previously [28]. The results are summarized in Fig 5. The data show that GC376 was most effective against FIPV 3CLpro by a FRET assay but it also substantially inhibited the activity of MERS-CoV and SARS-CoV 3CLpro.

## Discussion

Since FIP disease progression is quite rapid and the pathogenesis of FIP is primarily immune-mediated, an important question has remained unanswered as to whether antiviral drug treatment can effectively reverse disease progression in symptomatic hosts. It was previously shown that anti-inflammatory agent or antiviral immunity enhancing agents increased survival of mice infected with mouse-adapted SARS-CoV and treated with a NF-kB inhibitor [42] or various toll-like receptor agonists [31, 42–44], which was started shortly before or after virus infection. These reports indicate that controlling immune responses may prove an effective therapeutic strategy for coronavirus infections where inflammation plays an important role in pathogenesis. However, the available data on the efficacy of antiviral compounds failed to show sufficient effectiveness in mice infected with mouse-adapted SARS-CoV, even when treatment was started at the same time or shortly after virus infection [31, 43]. The observed low effectiveness of antiviral treatment is largely thought to be due to the use of compounds with weak anti-coronavirus activity and/or bioavailability. However, the lack of available potent antiviral compounds against coronaviruses has made it difficult to investigate the effects of antiviral treatment in animals with lethal coronavirus infection. Our 3CLpro inhibitors were previously reported to be potent against FIPV in the *in vitro* assays [28, 30] and effective at significantly reducing viral titers and tissue pathology in mice infected with MHV [30]. However, these 3CLpro inhibitors have not been tested in cats. In this study, a 3CLpro inhibitor, GC376, was determined to be safe with good bioavailability in cats. In the *in vivo* efficacy study using cats with FIP, the antiviral treatment started for cats at clinically advanced stages led to rapid normalization of the numbers of lymphocytes, during which time, fever, jaundice and ascites also resolved. The granulomatous lesions typically found in various organs in the cats infected with FIPV were not found or greatly reduced in the two cats that were euthanized after only 4 and 7 days of antiviral treatment. These results demonstrate that continuous virus replication is important in the progression of the immune-mediated pathogenesis of FIP and that controlling virus replication by a directly-acting antiviral compound targeting coronavirus 3CLpro is effective at reversing FIP disease. Our results provide the first evidence, to our best knowledge, that a direct-acting antiviral agent is effective at reversing the immune-mediated disease progression caused by coronavirus infection, even when antiviral treatment was started at clinically advanced stages. This finding may have important implication in devising effective therapeutic strategies for other coronavirus infections.

The conserved active site of coronavirus 3CLpro has been considered as a promising target for the design of broad-spectrum inhibitors for coronavirus infections [7, 45] and our group [28] and others [8, 46, 47] have previously reported the synthesis of 3CLpro inhibitors with antiviral activity against multiple coronaviruses. GC376 was previously shown to be active against FIPV in cell culture [28, 30] and SARS-CoV 3CLpro in a FRET assay [28]. In this study, we compared the activity of GC376 against the 3CLpro of FIPV, MERS-CoV and SARS-CoV by a FRET assay and determined that GC376 has most potent activity against the 3CLpro of FIPV. The IC_50_ values of GC376 against MERS-CoV and SARS-CoV were 2.16 and 4.87-fold, respectively, higher than that against FIPV in a FRET assay. These results indicate that GC376 is active against the 3CLpro of coronaviruses belonging to *alphacoronavirus* (FIPV) or the multiple clades in *betacoronavirus* (MERS-CoV and SARS-CoV), despite the low sequence identity of 3CLpro among FIPV, SARS-CoV, and MERS-CoV. The varying degree of effectiveness of GC376 against 3CLpro of different coronaviruses may reflect a subtle difference in spatial structure fit of the compound in the active site of coronavirus 3CLpro (S2B and S2C Fig). However, the antiviral activity of GC376 against the replication of these human coronaviruses has not yet been determined in cell culture or in the animal models.

A majority of reported protease inhibitors that are shown to have inhibitory effects against various coronaviruses in the enzyme assay are tripeptidyl or bulkier compounds [8, 48–51] and their antiviral activities in cell culture or *in vivo* properties are often not available. In this study, we compared the subcutaneous absorption of dipeptidyl and tripeptidyl 3CLpro inhibitors. GC376, a dipeptidyl compound, consists of a warhead, a Gln surrogate structure in a position that corresponds to the P1 position, Leu in the P2 position and a cap structure [28] (Fig 1). NPI64, a tripeptidyl compound, has homologous structural elements with GC376, except that NPI52 has an additional residue of 1-naththylalanine (Fig 1). These two compounds have comparable antiviral activity against FIPV in cell culture (Fig 1) [30]. However, the peak plasma concentration following a subcutaneous injection of NPI64 was considerably lower than that of GC376, which indicates that GC376 is absorbed better than NPI64 via the subcutaneous route. Our results on these closely related compounds indicate that relatively small structural change (addition of a residue) can have profound effects in absorption, and therefore the bioavailability of compounds needs to be taken into consideration early during drug selection process. GC376 was also found to be well-tolerated in cats during the 4-week duration of twice daily administration, with plasma drug concentrations remaining above the EC_50_ value but well below the CC_50_ value.

Emergence of viral resistance is a major concern in antiviral therapy. The only available literature on protease inhibitor-resistant coronavirus [52] reported that a 3CLpro inhibitor (GRL-001) has a low genetic barrier to MHV. In that study, resistant viruses were selected in 4 passage numbers in the presence of the inhibitor in cell culture, but the resistant viruses were highly attenuated in mice. To study the development of viral resistance against our 3CLpro inhibitors, we serially passaged FIPV in the presence of mock (vehicle), GC376 or the aldehyde form (NPI52) of NPI64. At passage 10, the EC_50_ value of NPI52 increased by 15-fold, compared to wild-type viruses passaged without NPI52, indicating the emergence of NPI52-resistant viruses. The 3CLpro gene of NPI52-resistant virus has a single mutation of S131C which located between the cII and dII strands in the domain II (S2A Fig). The role of this mutation in the 3CLpro in conferring resistance to NPI52 is currently not clear. However, the fact that serine at this position is conserved among all feline coronaviruses whose sequences are available and that C144, the active site residue that forms a covalent bond with the warhead of the inhibitor, is on the same loop as S131 between the cII and dII strands (S2A Fig) suggest that this mutation may influence the conformation of the loop and positioning of the active-site residue for proteolysis. Interestingly, Deng et al [52] also reported that one of the mutations on 3CLpro of MHV which is partially resistant to GRL-001 is located away from the catalytic site but in a position that may influence the conformation of the catalytic site. In contrast to NPI52, resistant viruses against GC376 have not been selected at up to 20 passages. These results indicate that similarly structured compounds may have different levels of resistance barrier against coronavirus 3CLpro. Interestingly, NPI52-resistant viruses did not lose susceptibility to GC376 in cell culture, indicating that this mutation did not confer cross-resistance. The mechanisms underlying differences in resistance development to these inhibitors need to be defined, but it may be speculated that the small size of GC376, compared to NPI52, makes it difficult for the virus to evade drug binding while retaining substrate cleavage capability. We are currently investigating the relative viral fitness of the resistant viruses and the role of the mutation in conferring resistance to NPI52.

In summary, a representative compound, GC376, of our dipeptidyl 3CLpro inhibitor series was shown to be safe by the dosage regimen used in cats and effective at reversing the progression of FIP even when the treatment was started at advanced clinical stages. Based on these results, this compound may have the potential to be developed into a safe and effective drug for FIP. Furthermore, broad activity of this compound against important human coronaviruses, including MERS-CoV and SARS-CoV, suggest that our inhibitor series may serve as a platform for further optimization for those important viruses. The results of this study also suggest that similar intervention approaches targeting virally-encoded 3CLpro warrant investigation for other existing and emerging coronavirus infections.

## Materials and Methods

### Experimental cats

Random bred cats free of most common feline pathogens, including feline enteric coronavirus, were obtained from the feline nutrition breeding colony, School of Veterinary Medicine, UC Davis.

### Ethics statement

All animal experiments were conducted in strict compliance with the Animal Welfare Act, PHS Policy and other federal statutes and regulations relating to animals and approved by the Institutional Animal Care and Use Committee at University of California, Davis (Protocol Number:17557).

### Synthesis of GC376

The syntheses of GC376 and NPI64 were previously described by our group [28, 53].

### PK and safety studies

In the single-dose PK study, two healthy SPF cats of 6–9 months of age (n = 2 for each compound) were subcutaneously injected with 10 mg/kg GC376 or 5 mg/kg NPI64 dissolved in 10% EtOH and 90% PEG400. Blood samples were collected from each cat at 2, 6, 12, 18, 24, 30, and 48 hrs following injection and plasma samples were prepared. The plasma drug concentrations were measured using routine high pressure liquid chromatography by Frontage Laboratories, Inc (Exton, PA). In the safety (multiple-dose) study, four healthy SPF cats of 6–9 months of age were injected s.c. with a daily dose of GC376 (10 mg/kg/dose dissolved in 10% EtOH, 50% PEG400 and 40% PBS) at 9AM and 5 PM for 4 weeks. Plasma samples were prepared for determination of drug concentrations at 2 or 16 hrs (immediately before the next dose) after drug administration for the first three days and weekly thereafter for 4 weeks. Measurement of plasma drug concentrations from the safety study was also performed by Frontage Laboratories, Inc. During the safety study, cats were monitored twice daily for adverse effects and body weight was measured daily. Prior to the first dose of GC376 and thereafter weekly, blood samples were collected from each cat for complete blood count and blood chemistry tests.

### *In vivo* efficacy study

A total of eight female or male SPF cats of 8–10 months of age in two independently conducted studies were originally part of another published experiment concerning the role of genetics in susceptibility/resistance to FIPV infection [54]. In that study, cats were inoculated with cat-passaged serotype I field strain FIPV-m3c-2. Among the cats that developed clinical and laboratory signs consistent with the abdominal effusive (wet) form of FIP and progressed to the point where they would be inevitably fatal, eight cats were transferred to the drug efficacy study. Four cats (P02, P03, P07 and P10) in the first study did not receive any medication other than GC376. Five doses of oral or subcutaneous meloxicam at 0.3 mg/kg/dose (once a day) as well as fluids were given to four cats (P15, P16, P17 and P24) in the second study for alleviation of pain and dehydration and discontinued before antiviral treatment was started. GC376 dissolved in 10% EtOH, 50% PEG400 and 40% PBS was given s.c. at 9 AM and 5 PM for 14–20 days. P03 and P07 were given GC376 at 5 mg/kg/dose for 4 or 8 days, respectively, and the dose was increased to 10 mg/kg/dose until the end of antiviral treatment. P15 and P16 were euthanized following antiviral treatment of 4 and 7 days, respectively. All other cats received GC376 at 10 mg/kg/dose during antiviral treatment. Animals were observed daily for clinical signs and body weight and blood was collected weekly for lymphocyte counts. Ascites were collected at multiple times before and during antiviral treatment from P15 and P16 for virus titration by real-time quantitative RT-PCR (qRT-PCR). The omentum samples were collected from P15 and p16 on necropsy.

### Virus quantitation by real-time qRT-PCR

Virus titers in the macrophages from the ascites and the omentum were determined by real-time qRT-PCR. Ascites (1 ml) collected from P15 and P16 were diluted at 1:5 in PBS containing 10 units/ml heparin. After centrifugation, the cell pellets were incubated with 500 μl RNAlater (Life Technologies, NY, USA) for overnight at 4°C. Cell pellets were then collected by centrifugation and stored at -20°C until analysis. Prior to total RNA extraction, 200 μl of PBS was added to the cell pellets. Omentum was cut into a size of less than 0.5 cm and placed in 5 volumes of RNAlater. Following overnight incubation at 4°C, samples were centrifuged for 5 min at 13,000 rpm to remove supernatant and tissues were stored at -70°C until analysis. Total RNA was extracted from the macrophages from the ascites and the omentum using RNeasy mini kit (Life Technologies) and real-time qRT-PCR was conducted. The primers and a probe targeting the 3’-UTR region of FIPV are 5’-GGAGGTACAAGCAACCCTATT-3’ (a forward primer), 5’-GATCCAGACGTTAGCT CTTCC-3’ (a reverse primer) and FAM-AGATCCGCTATGACGAGCCAACAA-Iowa Black (a probe). The relative levels of viral RNA in the samples were calculated by the comparative Ct method [39] using beta actin as a reference gene. The fold changes in viral RNA level in the macrophages in the ascites collected during antiviral treatment were calculated using the viral RNA level in the macrophage samples collected prior to the antiviral treatment.

### Serial passages of FIPV to generate viruses resistant to GC376 and NPI52

Sequential *in vitro* passage experiments using wild-type FIPV-1146 in the presence of GC376 or NPI52 were performed to select resistant viruses. Briefly, CRFK cells were infected with FIPV at an MOI of 0.05–1 in the presence of GC376 or NPI52 ranging from 0.5~3 μM. At each passage, supernatants containing viruses were passed on to fresh cells in the presence of GC376 or NPI52. Control mock virus was passaged in the absence of drug following the same procedure. Virus titers at certain passage numbers were determined by the 50% tissue culture infective dose assay and the fold changes in EC_50_ values relative to the wild-type virus were determined. After 10 passages in the presence or absence of NPI52, total viral RNA was isolated using the RNeasy Mini kit (Invitrogen) and the 3CLpro gene was sequenced following amplification by RT-PCR and analyzed for the presence of mutations. The viruses grown without the drug (mock) or NPI52-resistant viruses at passage number 10 were purified three times by limiting dilution [55].

To investigate if NPI52-resistant viruses are susceptible to GC376, serial dilutions of GC376 or NPI52 were added to confluent monolayers of CRFK cells in 24-well plates or cells were mock-treated, and the cells were immediately infected with NPI52-resistant virus at an MOI of 0.05–1. Following incubation at 37°C until an extensive cytopathic effect was observed in the mock-treated well (up to 36 hrs), cells were freeze-thawed for virus titration. The EC_50_ values were determined using Graphpad Prism software version 6 (GraphPad Software, San Diego, CA) following the procedures described previously [28, 30].

### Expression of 3CLpro

The codon-optimized cDNA encoding the full length 3CLpro of FIPV-m3c-2 was amplified by RT-PCR using the omentum tissue from the cats infected with FIPV-m3c-2. Those of SARS-CoV (GenBank: GU553365.1) and MERS-CoV (GenBank: KM210277) were synthesized by GenScript (Piscataway, NJ). The expression and purification of each 3CLpro were conducted following a standard method described previously by our group [28]. Primers for MERS 3CLpro are; forward primer (ATTCTAGAAAGGAGATATACCATGCAT CATCATCATCATCATAGCGGTCTGGTTAAAATGAGCC) and reverse primer (ATCTCGAGTCACTGCATCACAACACCCATAATC). Primers for FIPV 3CLpro are; forward primer (ATTCTAGAAAGGAGATATACCATGCATCATCATCATCATCATTCTG GATTGCGAAAAATGGC) and reverse primer (ATCTCGAGGCGGCCGCTCACTGACT).

### FRET assay

FRET assay was performed using a fluorogenic substrate (dabcyl-KTSAVLQ/SGFRKME-edans) derived from the cleavage sites on viral polyproteins of SARS-CoV [28] and was synthesized by GenScript. Methods for FRET assay were described previously by our group [28, 30]. Briefly, in the FRET assay, 3CLpro of FIPV, MERS-CoV, or SARS-CoV were incubated with GC376 for 30 min and the edans/dabcyl FRET substrate derived from the cleavage sites on SARS-CoV polyprotein was added to the mixture. Following incubation for 30 min, the florescence signals were measured and the IC_50_ was calculated for each 3CLpro [28–30].

### Three-dimensional structural model of 3CLpro

The structural model of FIPV 3CLpro was built based on TGEV 3CLpro (PDB ID: 4F49) [28] using the EasyModeller program (version 4.0) [56] and superimposed on the 3CLpro structure of MERS-CoV (PDB ID: 4WME, teal) [40]. The surface representation of the active sites of TGEV (PDB ID: 4F49) and MERS-CoV (PDB ID: 4WME) were created with PyMol (DeLano Scientific) [57].

## Supporting Information

S1 Fig Body weight and blood cell counts and blood chemistry measurements in cats subcutaneously administered with GC376 in the safety study. (A) Body weights of cats over time. (B-D) Various blood chemistry values or blood cell counts over time are expressed as means and standard error of the means. AP, alkaline phosphatase. BUN, blood urea nitrogen. ALT, alanine aminotransferase. AST, aspartate aminotransferase. ALP, alkaline phosphatase. Hg, hemoglobin.
(TIF)

S2 Fig Three-dimensional structural model of FIPV 3CLpro and surface representation of the active site of 3CLpro of TGEV and MERS-CoV. (A) Superimposition of MERS-CoV 3CLpro (PDB ID: 4WME, teal) [40] and FIPV 3CLpro (red) modeled using Modeller [56] based on TGEV 3CLpro as a template (PDB ID: 4F49) [28]. The 3CLpro of TGEV and FIPV are highly conserved with the amino acid sequence identity of >93%. Coronavirus 3CLpro forms a dimer for function but only the monomer form is shown here. The tan rectangle contains the active site located in the cleft between the domains I and II. The active site residues of 3CLpro of MERS-CoV and FIPV, Cys and His, are shown in orange and blue colors, respectively. The residue (S131) mutated in the 3CLpro of FIPV resistant to NPI52, an aldehyde form of NPI64, is shown in purple. (B and C) Surface representation of the active sites of 3CLpro of TGEV (PDB ID: 4F49)[28](C) and MERS-CoV (PDB ID: 4WME)[40](D). (B) The crystal structure of TGEV 3CLpro bound with GC376 (gray) in the S1 and S2 pockets of the active site of 3CLpro was previously published by our group [28]. The residues in the S1 and S2 pockets that form hydrogen bonds with GC376 are shown in yellow. (C) The S1 and S2 pockets of MERS-CoV 3CLpro are shown in pink. The residues that can potentially form hydrogen bonds with GC376 are indicated. All images were newly prepared using PyMol.
(TIF)
