# Correction: Reversal of the Progression of Fatal Coronavirus Infection in Cats by a Broad-Spectrum Coronavirus Protease Inhibitor

**DOI:** 10.1371/journal.ppat.1005650

**Published:** 2016-05-11

**Authors:** Yunjeong Kim, Hongwei Liu, Anushka C. Galasiti Kankanamalage, Sahani Weerasekara, Duy H. Hua, William C. Groutas, Kyeong-Ok Chang, Niels C. Pedersen

There are errors in the Funding section. The correct funding information is as follows: The in vitro assays and drug synthesis were supported by NIH grant R01AI109039 (http://www.nih.gov, PI: KC) and Morris Animal Foundation D14FE-012 (http://www.morrisanimalfoundation.org, PI: YK). The PK and safety studies were supported by Winn Feline Foundation Miller Fund MT13-006 (http://www.winnfelinefoundation.org. PI: NCP) and the Center for Companion Animal Health (http://www.vetmed.ucdavis.edu/ccah/. PI: NCP). The in vivo efficacy study was supported by the Center for Companion Animal Health (http://www.vetmed.ucdavis.edu/ccah/. PI: NCP) and the Kansas State University Global Food Systems program (GPRE002839) (PI: YK). Publication of this article was funded in part by the Kansas State University Open Access Publishing Fund. The funders had no role in study design, data collection and analysis, decision to publish, or preparation of the manuscript.
