# Supplementary figures and images for: Reversal of the Progression of Fatal Coronavirus Infection in Cats by a Broad-Spectrum Coronavirus Protease Inhibitor
Source: PLoS Pathog. 2016 Mar 30;12(3):e1005531. doi: 10.1371/journal.ppat.1005531 (PMC4814111; doi:10.1371/journal.ppat.1005531)

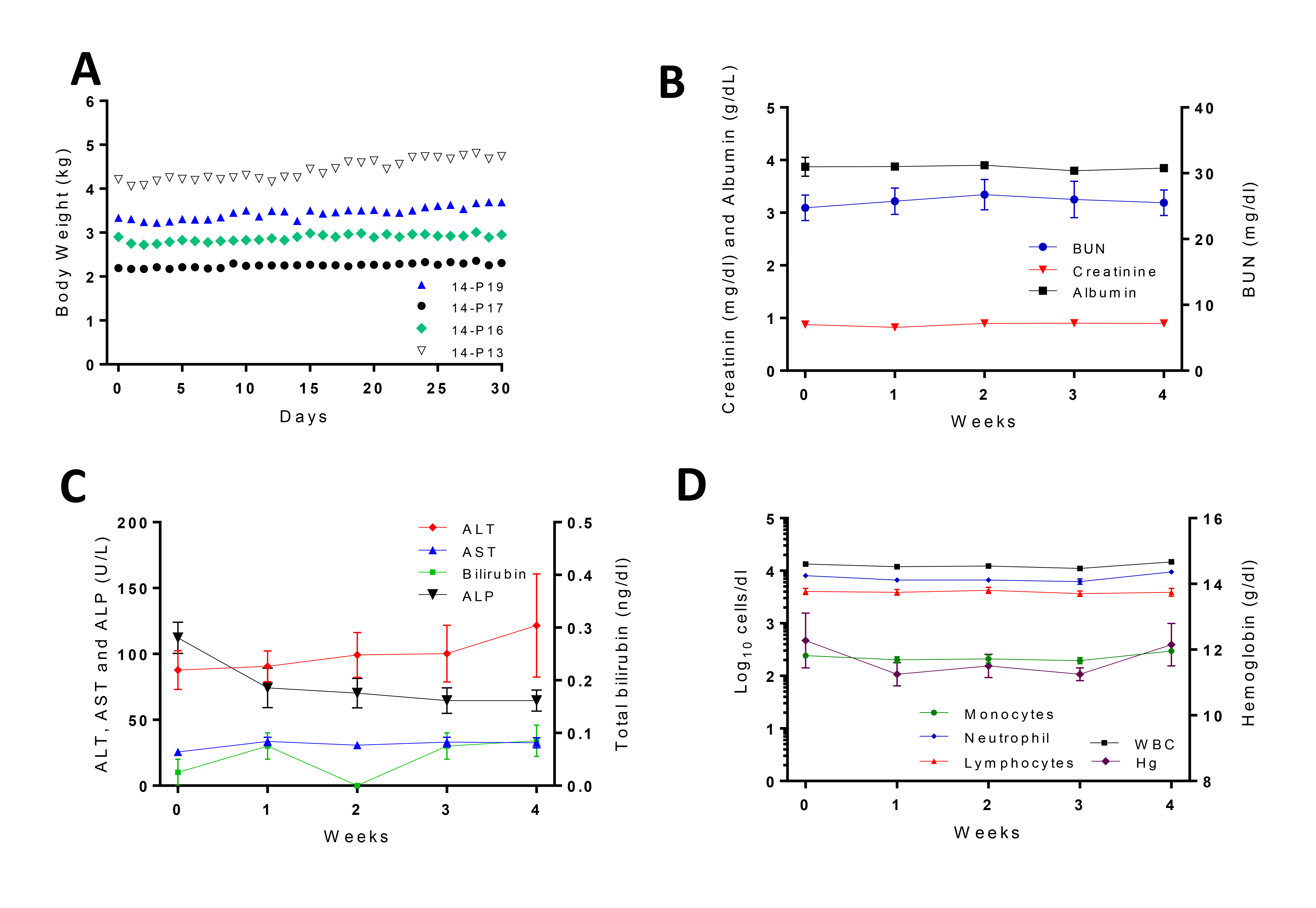

Supplement: S1 Fig — (A) Body weights of cats over time. (B-D) Various blood chemistry values or blood cell counts over time are expressed as means and standard error of the means. AP, alkaline phosphatase. BUN, blood urea nitrogen. ALT, alanine aminotransferase. AST, aspartate aminotransferase. ALP, alkaline phosphatase. Hg, hemoglobin. (TIF) [file ppat.1005531.s001.tif]

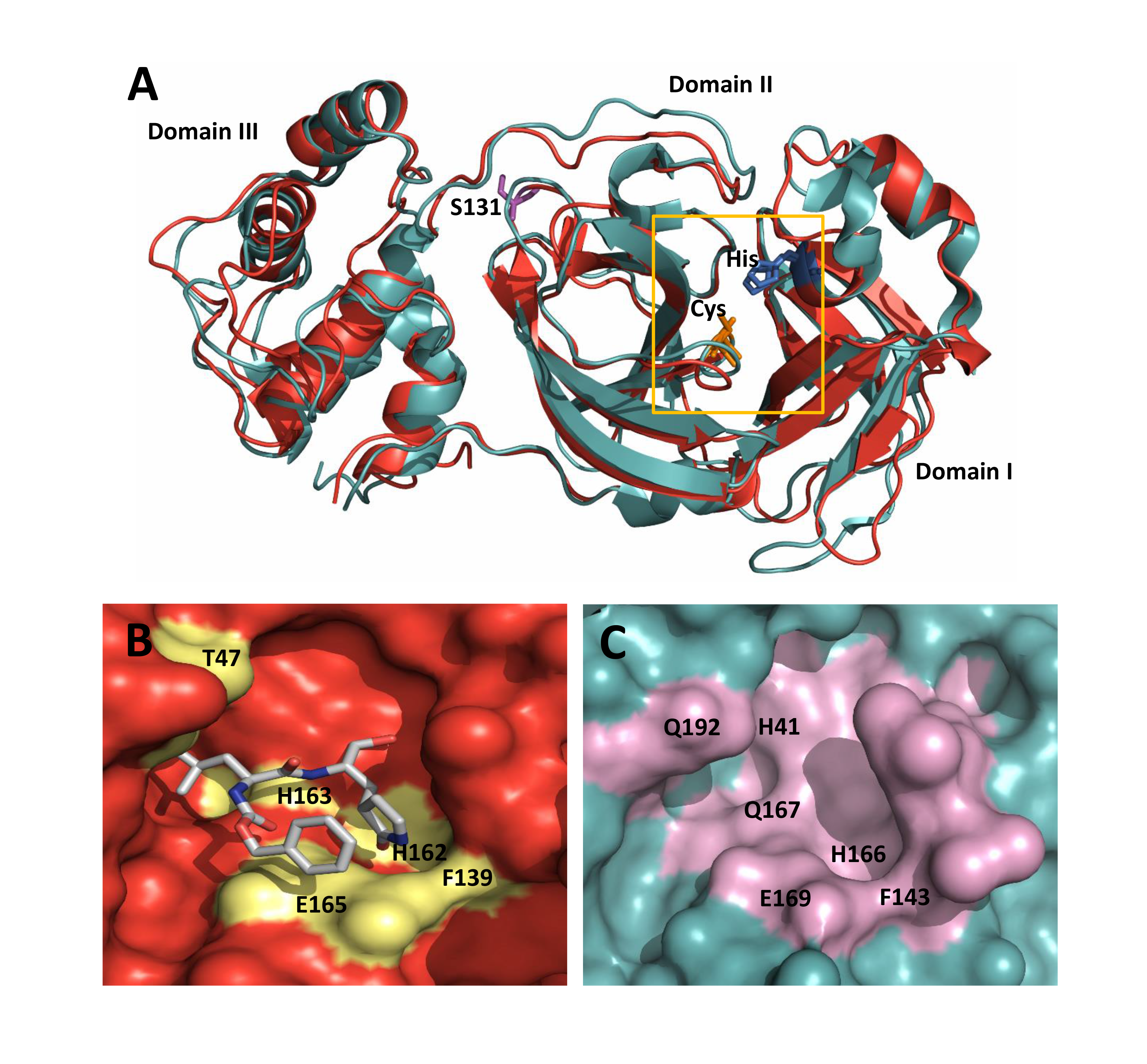

Supplement: S2 Fig — (A) Superimposition of MERS-CoV 3CLpro (PDB ID: 4WME, teal) [40] and FIPV 3CLpro (red) modeled using Modeller [56] based on TGEV 3CLpro as a template (PDB ID: 4F49) [28]. The 3CLpro of TGEV and FIPV are highly conserved with the amino acid sequence identity of >93%. Coronavirus 3CLpro forms a dimer for function but only the monomer form is shown here. The tan rectangle contains the active site located in the cleft between the domains I and II. The active site residues of 3CLpro of MERS-CoV and FIPV, Cys and His, are shown in orange and blue colors, respectively. The residue (S131) mutated in the 3CLpro of FIPV resistant to NPI52, an aldehyde form of NPI64, is shown in purple. (B and C) Surface representation of the active sites of 3CLpro of TGEV (PDB ID: 4F49)[28](C) and MERS-CoV (PDB ID: 4WME)[40](D). (B) The crystal structure of TGEV 3CLpro bound with GC376 (gray) in the S1 and S2 pockets of the active site of 3CLpro was previously published by our group [28]. The residues in the S1 and S2 pockets that form hydrogen bonds with GC376 are shown in yellow. (C) The S1 and S2 pockets of MERS-CoV 3CLpro are shown in pink. The residues that can potentially form hydrogen bonds with GC376 are indicated. All images were newly prepared using PyMol. (TIF) [file ppat.1005531.s002.tif]
